# How to Win a "House Job"
*Based on the Student Presidential Address, Galenicals, 1962/63.


**Published:** 1963-07

**Authors:** A. J. G. Dickens


### HOW TO WIN A "HOUSE JOB"*

BY

A. J. G. DICKENS

*Medical Student*

It is difficult to find an original subject that a student knows enough about to enable him to deliver an interesting lecture. I am not keen on non-medical topics for this Address, and I think there should be some connection with medicine, however attenuated it may be. I think my subject is relevant, judging by the number of anxious faces in the audience on the 25th of January! "How to win a house job" is not a subject to which there is a short answer, but rather one in which the importance lies in the summation of a lot of minor points plus a few more major and more definite ones, so I shall give a general factual background and then some more detailed advice.

The factual background I decided to obtain from a questionnaire that I sent to the the local consultants, after debating very seriously whether I dared do this.

Eventually I plucked up courage and did so. There was a very good response. I sent out 72 altogether, of which only 2 were to female members of Staff (both of these were returned), and I got back 13 on the very first day, including one from Professor Perry who wrote at the bottom "I have signed this as I am not ashamed of my views". I had requested anonymity on the questionnaires so that a full and frank opinion could be given, but I found this opening comment very encouraging. I received 12 on the second day, 4 on the third, 7 on the fourth, and eventually 50 came back within the first eleven days. I received 8 in the next thirty-nine days, including one during the week of the Address itself, so that the final number returned was 58 and this is 81 per cent of the total. 100 per cent of the obstetrical questionnaires were returned, as were 83 per cent of the surgical ones and 78 per cent of the medical ones. The replies indicated the general qualities liked by the consultants and you should think well upon them and try to apply them to yourselves where relevant. You cannot know too much about the subject, and the replies were fascinating in their variety and most worth while collecting. I think I shall keep all of them and, when rejected for my house job, I shall blackmail the consultant concerned, as I could identify by devious means the authors of two-thirds of the questionnaires!

### THE ANSWERS

*Question* 1 *asked what specialty the consultant worked in*, and I noted this myself when sending the questionnaires out.

*Question* 2 *asked "Do you prefer male or female housemen, or have you no special preference?"*

The replies to this were: "No preference" 27. "Male" 19. "Female" 2. "Best applicant regardless of sex" 2. A selection of other replies is as follows:—

"Female obviously".

"Female if beautiful and useful".

"Female because conscientious".

"Females tend to marry whilst men may practice locally and support your private practice!"

"The likelihood of temperamental clashes by female house surgeons with other

* Based on the Student Presidential Address, Galenicals, 1962/63.

women in authority in the hospital (Matron, Sisters, Almoner, Dispenser, Catering Officer) is greater than with male housemen".

"Whilst decoration and cheerful plumage help the day along, efficiency is more essential whatever the sex".

"No special preference but I like redheaded females".

"Male because easier to treat frankly".

*Question* 3 *asked "What are the important qualities of a houseman?"*

The selected replies were: "Conscientiousness" 11. "Common sense" 10. "Reliability" 10. "Unremitting hard work" 8. "Kindness to patients" 8. "A sense of humour" 8. "Enthusiasm" 8. "Intelligence" 7. "Some knowledge of the subject" 6. "Willingness to learn" 6. "Interest in the patients as well as the Chief" 6.(!) "Ability, especially to make quick decisions" 6. "Knowledge of one's own limitations" 4. "Attention to detail" 3. "Ability to speak and understand a little English" (!!) "Ability to picture himself as a patient in his own hospital". "Ability to give an accurate and detailed report on the state of any patient at any time". "Thoughtful treatment of the relatives". "Good manners", "Good handwriting", "Good humour", "Good temper", "Good with Sisters". "Blonde and of good vital statistics".

*Question* 4 *asked "How should the houseman dress*, (a) *male*, (b) *female?"*

The replies not differentiated by sex were: "Clean underneath white coats" 14. "Tidily" 11. "Neatly" 9. "Clean white coats" 5. "White coats on duty" 2. "Scrupulously clean white coat, buttoned up" 2. "Neatly and without eccentricity". "The aim is to look clean and this means changing your white coat occasionally". "No beards" 4. "I hate both men and women looking like dirty scruffy tramps". "Proper shoes". "Informal". "Fully clothed". "Not in the fashion of beatniks and teddies". A few notes on the male: "The houseman should not dress more expensively than I do". "No ostentation such as a beard". "Males should try to keep their hands out of their pockets at least 50 per cent of the time". "As he wishes, as long as his shirt is clean and his shoes are not of the winkle-picker style". And a few notes on the female: "No preference,—my wife won't let me". "Not in shorts, it's too distracting!" "Revealing". "Attractively and with good taste". "Attractively but not provocatively". "On the conservative side regarding make-up, as for many patients their problems are emotional". "No shorts, tights or bikinis". "Very like women". "No slacks or green or coloured stockings on duty". "As she wishes as long as she remembers that consultants are only human".

*Question* 5 *asked "Do you think it important that you like your housemen personally?"*

"Yes" 26. "No" 2, (physicians). "Yes, very important" 5. "Yes, emphatically". "Yes, definitely". "Yes, of course". "Yes, I hate beards". "Yes, a close relationship with understanding and respect". "Very". "Absolutely essential". "Imperative". "No, but desirable". "No, you get to like them if they are any good at the job". "Not essential but a very valuable asset". "Not vital, I like everyone (nearly)". "I always like them". "All doctors are so nice that this presents no difficulty".

*Question* 6 *asked "Should the houseman be gifted with social graces, or is professional competence sufficient?"*

"Professional competence sufficient" 16. "Prefer both" 14. "Both essential in order to enable him to treat all types of case really effectively, to give the patient confidence, and thus to ensure maximum co-operation". "He should be socially acceptable as no pre-registration houseman is likely to be professionally competent". "I do not mind about social graces but good manners help". "Not quite sure what this means—all doctors should acquire some bedside manners as it helps to deceive the patient". "The graces help but should not be excessive". "Preferably yes, as social graces make all intercourse easier, social, medical, etc.". "Social graces are part of the professional equipment". "I am not quite sure what social graces are. I think housemen should be house trained and understand that apple sauce when served with goose is not dessert".

*Question* 7 *asked "Should a female houseman ever be appointed in preference to a male one?"*

"Yes, if better candidate" 20. "Yes" 9. "No" 3. "At all times". "Sometimes". "Quite often". "Only under special circumstances". "I do not think so". "Don't understand the question". "Yes if she is a better man!" "Why not? A 'good' woman is surely better than a bad man!" "Yes, because by and large a female medical student of to-day is of higher quality both in brains and poise than a male". "Rarely in orthopaedics, as it is difficult to use orthopaedic know-how after marriage"(??). "Never, unless the male applicants were bad". "Yes, if her experience and qualifications are much superior and the male does not come up to a high standard". "Yes, if the rest of the staff are female, but never for a professional reason".

*Question* 8 *asked "Which sex has given you the better houseman on the whole?"*.

"Male" 19. "Equal" 16. "Female" 4. "I have had only two female house physicians both excellent, but one can never tell a female house physician what one really thinks of her". "The very best—all male; good average, either sex; bad, male more than female". "Females appear to do so because I am a male! They are not the weaker sex! They can tie knots as strong as any mere male" (surgeon). "Too complicated genetically and statistically for a mere Psychiatrist to answer". "Probably women as the competition is greater and so they are somewhat more carefully selected. Otherwise sex is irrelevant". "Both, but I like the sex to be definite in one direction" (mental health). "Male, but then no female house surgeon has ever proposed to me". "I am biased—I married mine!"

*Question* 9 *asked "Do you think it important if your houseman is married, and how does this affect his or her efficiency?"*

This question produced the greatest amount of comment of any of the twenty. On the whole marriage was thought undesirable, especially for the female who would have an impossible job to reconcile her simultaneous loyalties to home and to hospital, and very few Consultants indeed thought that marriage was anything other than harmful for her. For the male cautious optimism was expressed, and it was thought that marriage might be a good idea provided the houseman had a sufficiently well balanced personality to allow him to cope with a wife and a job. I will mention only three specific replies: "I prefer both sexes unmarried as they are not so anxious to get off duty, and their attitude to work is more single minded"; "In theory marriage may interfere with their work but in practice I have found it seldom does, and it may even be an advantage in my speciality" (obstetrics); "I prefer housemen not to be married. In radiotherapy the female houseman is inclined to worry about her gonads—or if she becomes pregnant *we* worry about them!!"

*Question* 10 *asked "Do you prefer a candidate for a higher post to have done house jobs in teaching or non-teaching hospitals?"*

"Teaching" 22. "Both" 18. "Non-teaching" 2. "Immaterial" 6. "You cannot get a higher post if you have not done a house job in your own teaching school, otherwise do both". "Teaching hospital appointments have a mysterious advantage". "If he has had a job at his own teaching hospital it is useful, but it is only necessary to do one". "He must have done jobs in both, and I would be unlikely to appoint to Registrar or higher if he has not done a teaching hospital job". "Both, the first for traditional reasons and the second for real work". "With whom he has worked is of far more importance than where".

*Question* 11 *asked "Did you obtain your first choice house jobs when you applied for them?"*

"Yes" 38. "No" 12. "Of course!" "Certainly". "In my day one was invited". "Cannot even remember!" "I turned mine down, but it was during the War".

*Question* 12 *asked "How many house jobs did you do?"*

Replies summarised:—

| | |
|---|---|
| 1 house job, | 2 consultants. |
| 1½ house jobs, | 1 consultant. |
| 2 house jobs, | 6 consultants. |
| 3 house jobs, | 11 consultants. |
| 4 house jobs, | 14 consultants. |
| 5 house jobs, | 6 consultants. |
| 6 house jobs, | 9 consultants. |
| 7 house jobs, | 2 consultants. |
| 8 house jobs, | 1 consultant. |
| 9 house jobs, | 2 consultants. |

Many people stressed the effect of the War on their house appointments, and no very profound conclusions can be drawn from these general totals.

*Question* 13 *asked "What qualities did you most admire in your Chiefs?"*

"Competence and ability" (especially in surgery) 20. "Intellectual honesty" 11. "Ability to teach" 9. "Kindness to patients and staff" 9. "Brilliance in clinical judgment and work" 6. "Interest and concern for patients and juniors" 6. "Sense of humour" 4. "Humanity" 4. "Wisdom" 4. "Appreciation of good work done by those under them" 3. "Humility in error" 2. "Imperturbability" 2. "Ability for gauging accurately what I could do on my own". "Ability to opt out of the rat race—I fear for my coronaries!" "Ability to delegate to their juniors". "Ability to inspire". "A capacity to admonish when necessity demands it and to congratulate when occasion suggests it". "Bedside manner—this included convincing the patient that (a) the best man was looking after him, and (b) he was certain to get better as a result". "No comment, most of them bored me". "Cannot think of any".

*Question* 14 *asked "Are you now in the specialty you first wished to enter?"*

"Yes" 41. "No" 15. "Yes, but I intended general practice"(!!). "Yes, although I sometimes wish I had gone into general practice and had a profit". "No, would still prefer to be a physician at times" (surgeon). "I flirted with gynaecology but gave it up because I wanted to be a surgeon". "Slid into it on account of wife's higher qualifications" (surgeon).

*Question* 15 *asked "If intending to specialise, do you think one's pre-registration jobs should be in that specialty?"*

"Yes" 3. "No" 51. "No, unless it is merely a trial run in the specialty to make sure that it is really what you want to do". "Certainly—you may get disillusioned". The value of general medicine and general surgery for the pre-registration jobs was very heavily stressed by almost everyone.

*Question* 16 *asked "If intending to specialise, do you think one's post-registration jobs should be from as widely differing a range as possible or should specialisation start already?"*

54 favoured a wide range, 4 were against it. This question produced the strongest body of similar opinion on any one subject, as no less than 54 out of the 58 consultants said the same thing. The only occasion on which the wide range was thought unnecessary was where the houseman could apply for jobs in a broadly similar subject to the one he eventually intended. "Post-registration jobs should be in subjects akin to but not in the chosen specialty, so that one is knowledgeable on the borderlines where one specialty may overlap with another".

*Question* 17 *asked "If entering general practice, which specialist house jobs are most useful for the future?"*

Obstetrics, 33. E.N.T., 25. Dermatology and paediatrics, both 21. Gynaecology, 15. Casualty, 10. Ophthalmology, 7. Psychiatry, 6. Orthopaedics, 3. "Medicine so as to learn to use a stethescope, surgery in order to be able to diagnose the acute abdomen, and psychiatry so that you will know something about life" (mental health).

And from one physician:—

"(1) An internship in the Stock Exchange—Bristol or London.
(2) Membership of Failand Golf Club.
(3) Paediatrics.
(4) Geriatrics.
(5) Mediatrics."

*Question* 18 *asked "Which jobs would you have liked to do, which you were unable to do?"*

None, 18. General medicine 14. E.N.T., 5. General surgery, paediatrics, orthopaedics, 4. Thoracic surgery, plastic surgery, 3. Ophthalmology, dermatology, hearts, chests, obsterics, gynaecology, neurology, neurosurgery, 2. "Not another one—I wanted to get on and earn my living". "None—I was jolly good at getting jobs"(!). "Dermatology, because no emergencies". "House Physician to Dr. Gillespie or Ben Casey".*

*Question* 19 *asked "What would you say the houseman learns from his house jobs?"*

"To take responsibility" 11. "Practical experience in medicine and surgery" 10. "That he is really still a student learning to practice what he has been taught in theory" 8. "To deal with patients, relatives and staff" 8. "How to treat people, not just disease" 7. "To manage the patients, examine, diagnose and treat them as he thinks right" 6. "Self-confidence" 6. "To help in a team" 5. "To recognise serious illness, and when it changes for the worse" 3. "How little he knows" 3. "The gentle art of filling up forms and signing certificates" 2. "The hospital's relationship with all the outside agencies—Police, Mental Health, Social Services, etc.", 2. "That qualification is the beginning and not the end". "To weed out the neurotics and the compensation neuroses". "All his Chief's failings". "That it is all so different from the books". "That commonest things are commonest". "That he knows very little, and that he should not be in a hurry to utilise too vigorously those snippets of knowledge which he thinks he has". "No one knows anything when he qualifies. It is only when faced with a patient who needs treatment that he learns". "Many get hydrocephalic in these early months and are finished as far as I am concerned". "Intimate contact with his patients, and ability to organise his operating lists—a really capable House Surgeon can even organise his Senior Registrar and Chief. In consequence he learns his clinical medicine and surgery, but he is wasting his time and not getting the best out of his house job if at the same time he is trying to work for a higher examination".

*Question* 20 *asked for "Any further comments"*:—

"The important thing to remember about pre-registration jobs is that they are post-graduate teaching appointments. Primarily they are meant to enable the graduate to continue his medical education, and they should provide time for this responsibility. A large number of patients, with the so-called clinical experience which this provides,

* Ref. contemporary television programmes. (Ed.)

is therefore fundamentally bad in a pre-registration post. Such posts should be arranged differently".

"The creeping-in of the registrar class between the Surgeon and his House Surgeon has caused the greatest change in my time, and must have grieviously affected the post of House Surgeon".

"What benefit a houseman derives from the job depends mainly on his own character and efforts. He should feel that he is fairly hard worked but that this is a help to him to make himself a better doctor, and he should enjoy his residence".

"Avoid a job which is known to be too busy—you get into bad habits".

"I think most strongly that for an embryo doctor to develop his presumed ambition to become a *good* doctor he should realise the importance of a broad education. By this I mean not only from the academic, but also from the humanitarian and philosophical, viewpoints. Only if he is a really 'balanced citizen' can he be the most effective sort of doctor and have the best prospect of doing most good to the 'common weal' ".

"A period of research or in close association with it, is very valuable for any medical career; almost any sort of research, but preferably orientated towards the future career".

"The best way of winning a house job is to impress throughout your clinical training your Professor and Lecturers with your keenness, reliability and '*horse sense*' ".

"A career in medicine or surgery is often governed by accident rather than design. To take every opportunity, and always to act as if dedicated to whatever job in which one finds oneself at that particular moment, is the way to get on. It is a rat race, and it is the other rats rather than the traps or the cats that matter. It is every rat for himself!"

"When you are making application for any post, remember that you are temporarily in the position of someone who has something to sell. Without over-doing it, 'sell yourself big'. Take trouble over the way in which you set out your application form; do not cram all the information into the smallest possible space; even go to the length of purchasing really good paper for the purpose. Competition is keen, and attention to these small details can tip the balance".

"If you want a house job you will get it by seeking an interview with the appropriate consultant early. This is polite and mildly flattering. The haircut, neat suit, and clean hands help at the interview".

"A House Surgeon works very hard, and therefore tends to play hard when off duty. Proximity to the opposite sex (nurses, etc., and medical) probably accounts for the high marriage rate between likes".

"I hope I have passed!"

"And the best of luck".

"Good luck with your 'house hunting' ".

"You ought to get a house job!"

I am most grateful for all the interest shown and the trouble taken by the consultants without whose co-operation these very useful facts and opinions would not be known. More specifically I have asked the advice of two senior staff members and I will pass on what they said.

The first one thought that he tended to appoint "Chaps I remember". The Firm Reports consist of marks for diligence and ability whilst on the Firm and these are looked at by the appointing committee, particularly to see their own marks originally given to the candidate. He stressed two important adages—"As was the student, so is the doctor" and "I want him, I remember him". To have done a *locum* on the Firm concerned is also a good recommendation, providing it was done well and you are remembered with pleasure and not horror. He also emphasised the importance of doing house jobs in your own teaching hospital, and this is always checked upon when you apply for higher posts. After all, if your own teaching hospital did not appoint you to a single one of its house jobs then the inference is that there must be some very good reason why it did not do so, and this fact can count against you when a candidate for a higher post. Plans are well advanced for trying a scheme of rotating jobs at S.H.O. level: these would last one year and consist of three 4-monthly appointments chosen amongst others from E.N.T., orthopaedics, casualty, ophthalmology, dermatology, paediatrics, and mental health. The number of beds per houseman is to be reduced to about 20 to 25 to enable him to have time to work properly for his patients' and his own benefit.

The second staff member also stressed how essential it was that the early jobs should be done in the teaching hospitals—"Many students forget this or do not know it". With a record behind you of a teaching hospital house job you have not committed yourself and you can still apply for higher posts or not, as you wish.

### SOME COMMENTS

From the replies, I think it is significant that only two consultants definitely preferred female housemen, and that forty-six preferred males or had no set choice. As a male, I find this encouraging, but in fact the desire was for the best houseman, and males were thought to be the safer average choice. There was only one mention of males being chosen for reasons of physical strength—"Thoracic surgery, involving very many operating sessions, is too exhausting for the average female". That only one consultant should mention this surprised me, as I had anticipated more support for this view, especially among the surgeons.

"He should remember that the hospital motto is 'Charity Universal' ", and "He should show courteousness to his juniors as well as to his seniors" were two comments on the attributes of a houseman that I thought most appropriate, and having recently been a patient in the B.R.I., I really agreed with "The ability to picture himself as a patient in his own hospital". Being a patient was very interesting, and I saw life from quite a different angle. A spell on the receiving end makes one realise how important it is that the houseman should be of "good" character.

I thoroughly agree with two of the answers on dress, *viz.* "A casual or untidy appearance, rightly or wrongly, may lead patients to assume that the same low standards may be applied to professional work", and "They should dress cleanly and smartly, bearing in mind the effect that their appearance will have on the patients they will be treating". I am appalled by the unprofessional standard of some of the students regarding dress—coloured stockings, stiletto heels, shapeless sweaters, too exaggerated hair-styles, beards and moustaches, continental ties, dirty trousers or even jeans, "winkle-pickers" or scruffy "creepers"—these are all far too common, and should not be seen at all on a student on duty. Perhaps I am already old-fashioned, but I am sure that the average standard of dress has gone down even while I have been at Bristol.

I am surprised that two consultants did not think it mattered whether they liked their houseman or not, and that five more thought it desirable but not essential that they should so so. I agree with—"Certainly they should, as a positive dislike is hard to bear for six months", and "Yes, because trust in judgment is influenced by liking a person". The houseman usually likes his chief, and this is a factor in choosing his job; and if the feeling is mutual good results are much more likely to come forth.

Forty-two consultants thought that social graces were part of professional equipment, and this question produced the most comment except for the vexed subject of marriage. Most answers were very gracefully expressed (perhaps showing some of these same social graces). Generally, professional competence *on its own* was thought to be less essential. Some of the most usual sentiments were—"Good manners, consideration for others, sympathy, and wit", "A sympathetic nature and an ability to communicate", "Professional competence alone is quite inadequate for any clinician". All students should try to develop these qualities.

Regarding female housemen, potential marriage seems the predominant drawback. "Sex should not control such decisions, but one can't help remembering that training a man is more profitable, as he will continue as a doctor"; and another comment was "Too much wastage among the girls through marriage". Indeed there were many reservations about choosing females as housemen. Women were thought to be of higher critical standard, on average, when starting medicine, and this advantage persisted; they were more conscientious, but also more liable to get on the wrong side of the nursing staff; in appointments their sex-appeal weighed heavily in their favour.

This brings us back to the question of marriage. It was thought suitable for male housemen only, and they had to be mature enough to cope with home and hospital, which would be too difficult a task for most female housemen. I think that the marriage partner of a houseman gets an even worse time, especially if working non-medically, or looking after a flat or family. I have a cousin who married a male houseman who did two very busy jobs, and she always regretted not waiting until after the house year before trying to set up a home. If you wish to get married as a student, then that is your own decision, but if not, then wait at least until Registration, and do not marry to celebrate Graduation.

One question asked consultants what qualities they most admired in their own previous Chiefs. Answers that particularly appealed to me were—"Ability to teach", "Sense of humour", "Imperturbability", "Quotability", "Devotion of their patients to them", "Simplicity in explanation", "Social approachability", and "Their human failings". I think your Chiefs remain a definite and lasting memory of your training, and that subconsciously you copy some habits from them. But try to copy the better ones! I noticed that only one consultant mentioned regular attendance, and none mentioned punctuality as admirable qualities in their own Chiefs. Had the others assumed that these were inseparable from the status of a Chief, or had they perchance entirely overlooked them?

Today, with two house jobs obligatory, and the possibility that in the future rotating S.H.O. jobs in the specialties that I have mentioned may be added to them, the situation cannot arise whereby 14 consultants (including 2 physicians) had occupied no post in general medicine, and 4 (including 2 surgeons) had occupied none in general surgery. It is difficult to strike the balance between obtaining a wide range of post-graduate experience and starting to specialise, and advice from your Chief is always valuable, and should be asked for.

What do you learn during your house jobs? Some of the answers were—"Endurance", a "Basic training for the future", and, more importantly, "To be a good doctor, as opposed to being an expert at examinations". There are two further opinions, which I should like to leave with you as well worthy of your serious consideration. I quote first a physician, and then finally a paediatrician:—"The first and second house jobs ought to be the high-light of a doctor's career—the most exciting and dramatic year of his life. Too often today you find housemen bellyaching about pay or conditions while at the same time evading responsibility when it is offered to them. This is a pity. Perhaps it is the times we live in, perhaps the fact that a houseman has so many bodies senior to him also interested in the patient, that he is forced into the position of a dogsbody. He doesn't need to allow this to happen, but it often does." And "The undergraduate medical course is only interested to teach the scientific saws of medicine, and the practical methods required to apply them, in general terms.

It is *not* intended to train the student for any particular type of medical life subsequently. The hospital junior jobs are intended to teach the graduate how to become a practical doctor, and acquire the special skills required for the discipline of whichever branch of medicine he chooses ultimately to practise".

I have not altered any part of the consultants' opinions that I have quoted, and I think that therein lies the value of my lecture, as you do not have to take my word for any of it, but you may read what your Chiefs actually think.

So, to sum up, the following are the more major and more definite points about winning a house job:—

1. It is essential to get a good reputation on the Firm. Everything you do in the hospitals is noticed, and will be remembered by someone. You cannot be too careful about your actions on the Firm, and you cannot start too soon on your attempt to convey a good impression of yourself.
2. Always dress to a professional standard. The patients take a lot of notice of your dress, and it is certainly remembered by members of the staff whether a student looks civilised or not.
3. Appear on the senior rounds of the Firm for which you might apply. This is very tactful, and of benefit to you in all sorts of ways, especially in acquainting you with the idiosyncrasies of the Chief.
4. Do the relevant *locum* if at all possible. This may not teach you very much about the subject concerned, but it will teach you to be a reasonable administrator (which is what most housemen are required to be).
5. Apply early and carefully for your chosen house job. Aim at one you have a good chance of getting, and know who are your rivals for the post.
6. Be grateful for the appointment, and make the staff think that after all you have only done the right thing in applying for it.
7. Work very hard—you need the reference for your future jobs.

You cannot know too much about house jobs, and the winning of them does really reflect the opinion already held of you after three years in the hospital. Start thinking of this when you pass second M.B., and even more so as you lead up to third M.B. I have probably lost any chance I ever had of getting a house job in Bristol, but may I wish you the very best of luck when you apply for yours!

